# What supports physiotherapists’ use of research in clinical practice? A qualitative study in Sweden

**DOI:** 10.1186/1748-5908-8-31

**Published:** 2013-03-14

**Authors:** Petra Dannapfel, Anneli Peolsson, Per Nilsen

**Affiliations:** 1 Department of Medicine and Health, Linköping University, Linköping, SE 581 83, Sweden

**Keywords:** Physical therapy, Evidence-based practice, Research use, System levels, Attitudes, Clinical practice

## Abstract

**Background:**

Evidence-based practice has increasingly been recognized as a priority by professional physiotherapy organizations and influential researchers and clinicians in the field. Numerous studies in the past decade have documented that physiotherapists hold generally favorable attitudes to evidence-based practice and recognize the importance of using research to guide their clinical practice. Research has predominantly investigated barriers to research use. Less is known about the circumstances that actually support use of research by physiotherapists. This study explores the conditions at different system levels that physiotherapists in Sweden perceive to be supportive of their use of research in clinical practice.

**Methods:**

Patients in Sweden do not need a referral from a physician to consult a physiotherapist and physiotherapists are entitled to choose and perform any assessment and treatment technique they find suitable for each patient. Eleven focus group interviews were conducted with 45 physiotherapists, each lasting between 90 and 110 minutes. An inductive approach was applied, using topics rather than questions to allow the participants to generate their own questions and pursue their own priorities within the framework of the aim. The data were analyzed using qualitative content analysis.

**Results:**

Analysis of the data yielded nine favorable conditions at three system levels supporting the participant’s use of research in clinical practice: two at the individual level (attitudes and motivation concerning research use; research-related knowledge and skills), four at the workplace level (leadership support; organizational culture; research-related resources; knowledge exchange) and three at the extra-organizational level (evidence-based practice guidelines; external meetings, networks, and conferences; academic research and education).

**Conclusions:**

Supportive conditions for physiotherapists’ use of research exist at multiple interdependent levels, including the individual, workplace, and extra-organizational levels. Research use in physiotherapy appears to be an interactive and interpretative social process that involves a great deal of interaction with various people, including colleagues and patients.

## Background

The need for a more research-informed physiotherapy practice was recognized decades ago, yet the issue did not receive high visibility until the emergence of the evidence-based practice (EBP) movement in the 1990s. Research use is considered an important aspect of EBP, which has been defined as ‘the conscientious, explicit and judicious use of current best evidence in making decisions about the care of individual patients’ [1]. Other definitions of EBP have a wider perspective that encompasses the views of patients and clinicians’ experience-based knowledge for clinical decision-making alongside the role of research evidence. Since the late 1990s, professional organizations have identified EBP as a priority and influential researchers and clinicians have argued that physiotherapists have a moral and professional obligation to base their practice on research findings and move away from techniques based on anecdotal testimonies or opinion [2-9]. However, concerns have been raised about some aspects of EBP, including the usefulness of randomized controlled trials to provide clinically applicable physiotherapy evidence and the low priority afforded qualitative research in the evidence-based hierarchy of evidence [10-16].

Numerous studies in the past decade have documented that physiotherapists hold generally favorable attitudes to EBP and recognize the importance of using research findings to achieve a more evidence-based clinical practice [17-24]. However, there are many challenges and barriers to physiotherapists’ research use, including time restrictions, limited access to research studies, poor confidence in skills to identify and critically appraise research, and inadequate support from colleagues, managers and other health professionals [17-23,25-27]. The barriers to EBP in physiotherapy are largely similar to those pertaining to other healthcare professions [23,28]. There is also a paucity of research in some areas of physiotherapy, which constitutes an obstacle to practicing evidence-based physiotherapy [4,13].

Despite the fact that research in several fields has identified barriers to research use at different system levels, from the individual to the organization, interventions to achieve a more EBP have predominantly targeted individual healthcare professionals to influence their attitudes, beliefs, knowledge, and skills as a means of changing clinical practice [28,29]. It has been argued that implementation research has ‘failed to fully recognize or adequately address the influence and importance of healthcare organizational factors’ [2,30]. However, the importance of contextual conditions for use of research in healthcare has increasingly been recognized [31-36].

This study addresses important knowledge gaps concerning the use of research to guide physiotherapy practice. While barriers to research use are fairly well established in previous research, it is not self-evident that the removal or reduction of these barriers results in increased use of research in clinical practice. Hence, it is important to investigate the circumstances that physiotherapists have found to actually support their use of research findings in routine practice. Using focus group interviews, the aim of this study was to explore the conditions at different system levels that physiotherapists in Sweden perceive to be supportive for their research use in clinical practice.

## Methods

### Study setting

This study took place in Sweden. Healthcare in Sweden is publicly funded, *i.e.*, residents are insured by the state, with equal access for the entire population and fees regulated by law. The provision of healthcare services is the responsibility of the 21 county councils in Sweden [37]. There are approximately 21,000 authorized physiotherapists in Sweden [38]. They are employed by county councils (public sector), occupational healthcare organizations (private or public sector), or work in private organizations, as employers or employees (private sector).

Patients in Sweden do not need a referral from a physician to consult a physiotherapist and are free to choose a physiotherapist from the private or public sector. Physiotherapists in Sweden have a great deal of autonomy. They are entitled to choose and perform any physiotherapeutic treatment technique they find suitable for the individual patient.

### Study design and participants

A qualitative approach with focus group interviews was used to investigate Swedish physiotherapists’ perceptions of conditions that support research use in clinical practice. The aim of focus groups is to explore experiences, attitudes, and ideas concerning a specific set of issues in a given cultural context [39]. The group dynamic of focus groups can facilitate the participants’ discussions and reflections as they listen to one another’s opinions, potentially generating new insights, ideas, experiences, or perspectives about the topic that might not arise in individual interviews.

Eleven focus group interviews were conducted from March to June 2011 involving 45 physiotherapists from five county councils in Sweden. Participants for the focus groups were recruited through managers and other key people in different clinical settings in Sweden via an e-mail in which the study was briefly described. The request was sent to 50 hospitals, primary care units, and private physiotherapy clinics. All who answered positively were asked to invite physiotherapists in their department, unit, or clinic to participate in the study. They were encouraged to invite whole teams of physiotherapists to avoid bias due to selection of specific physiotherapists. Each focus group consisted of physiotherapists from the same workplace, but they did not necessarily work as part of the same team although they shared the same management. The focus groups included physiotherapists of different seniority, educational degrees, and age. In order to encourage free responses, none of the managers of those participating in the focus groups were present.

A purposeful selection approach was used to achieve a heterogeneous sample of physiotherapist groups, which represented a broad spectrum of experiences and contexts to strengthen the validity of the study [40]. Variety was sought according to clinical setting, geographic location, the number of years of practice, and educational levels (Table 1). The study was approved by the regional ethical review board at Linköping University, Sweden.

**Table 1:** Sociodemographic data of the focus group participants (N = 45)

**Characteristic**	**Value**
Demographics	
Gender, n (%) female	33 (75)
Age, mean years (SD)	41 (11); range 22–62 years
Practice and education	
Years of practice, mean years (SD)	13 (9.2); range 1–37 years
Years of education, mean years (SD)	2.9 (0.5); range 2–5 years
Masters degree, n (%)	2 (4.4)
Bachelor degree, n (%)	31 (69)
Courses beyond the basic physiotherapy education, n (%)	45 (100)
Participated in non-academic courses, n (%)	37 (82)
Participated in academic courses, n (%)	29 (64)
Employment	
Part-time employee, n (%)	9 (20)
Full-time employee, n (%)	36 (80)
Location of the unit (N = 11)	
Rural setting, n (%)	4 (36)
Urban setting, n (%)	7 (64)
Type of unit (N = 11)	
Hospital setting, n (%)	6 (55)
Primary care, n (%)	3 (27)
Private clinic, n (%)	2 (18)

### Data collection

An inductive approach was applied in the study, using topics rather than questions to allow the participants to generate their own questions and pursue their own priorities within the framework of the aim. The topics were developed by the authors of the study and were scrutinized in a seminar with ten physiotherapists, most of whom combined research with physiotherapy practice. In addition, four physiotherapists who took part in the seminar participated in a pilot focus group before the interviews were carried out; this pilot interview was not included in the study due to the participants’ experience of conducting their own research.

Each focus group interview began with an open question asking the physiotherapists to describe their work and workplace. The interview then focused on four topics: perception and experience of research use; organizational routines and/or structures supportive to research use; organizational conditions that support, enable, or facilitate research use; and collaboration with organizations conducting research.

The focus group interviews were conducted during regular working hours to facilitate participation. Each focus group interview lasted between 90 and 110 minutes. Before the start of the interview, the participants filled in a questionnaire with a few background questions (Table 1).

The participants were informed of the confidentiality of their contribution, that participation was voluntary, and that they could withdraw at any time during the interview. Two moderators attended all focus groups except for one interview. The first author of this study led the interviews and asked follow-up questions. The second moderator took field notes and made observations. The information recorded by the second moderator was used to discuss interpretations of the interview with the interviewer if there were discrepancies or lack of understanding of what was said. In general, discussions in the groups were fluent and little steering from the moderator was needed. The open climate encouraged everyone to express their opinions.

### Data analysis

Interviews were recorded on tape and later transcribed verbatim by the first author. The data were analyzed using qualitative content analysis in accordance with Krippendorff [41]. Content analysis is a technique for analyzing texts based on empirical data with an explorative and descriptive character, and entails a structured analysis process to code and categorize the data [41].

The focus group interviews were analyzed in several steps. Each author read all the transcripts to obtain an understanding of the whole. The first author reviewed the transcripts and indentified coding units in the text that captured various key statements and thoughts in relation to the study aim. All the researchers scrutinized the coding units and reviewed the text several times. During this process, the coding units were merged into context units by the three authors. The context units included several coding units and reflected more than one key statement or thought. The context units were combined into categories based on similarity of the content by the three authors. These categories were based on conditions that the focus group participants mentioned as being supportive to research use. The categories were merged into three overarching system levels based on their characteristics by the three authors.

During the process, all authors discussed the content of the categories using triangulating analysis, *i.e.*, the authors independently analyzed the same data and compared their findings. The discussions continued until no inconsistencies existed and a shared understanding was reached to prevent researcher bias and strengthen the internal validity [40]. Quotations were identified to report the findings and illustrate the content, and were translated from Swedish to English.

Research use was interpreted in the analysis in accordance with well-established definitions. We accounted for both instrumental research use (changes in the physiotherapists’ practice based on research findings), and conceptual research use (changes in their understanding, knowledge, and attitudes), which reflects changes in thinking rather than actual behaviour [42].

## Results

Analysis of the data yielded nine categories that the participants discussed in relation to conditions that have supported or facilitated research use in their clinical practice. These categories corresponded with three overarching system levels (Table 2).

**Table 2:** Overview of the results: system levels, categories and explanation of the categories

**System level**	**Categories**	**Explanation**
Individual level	Attitudes and motivation concerning research use	Positive attitudes and motivation to use research in clinical practice are supportive to research use
	Research-related knowledge and skills	Knowledge and skills for tasks such as appraising research studies and assessing the strength of evidence are supportive to research use
Workplace level	Leadership support	Formal and informal leadership support and directives on a research-informed clinical practice are supportive to research use
	Organizational culture	An organizational culture that fosters learning and competence development is supportive to research use
	Research-related resources	Availability of various resources, such as access to research, time, and financial and personnel resources, is supportive to research use
	Knowledge exchange	Knowledge exchange with other clinicians and patients is supportive to research use
Extra-organizational level	EBP guidelines	Availability of various guidelines to assist decisions about appropriate treatment is supportive to research use
	Involvement in external meetings, networks, and conferences	Discussion and interaction with external physiotherapists on research matters are supportive to research use
	Involvement in academic research and education	Interaction and engagement with research and teaching activities are supportive to research use

### Individual level

#### Attitudes and motivation concerning research use

The physiotherapists believed that having positive attitudes to research and a strong motivation to use research in clinical practice provided favorable conditions for research use. However, they noted that interest in research varied among their colleagues:

‘Some are really into this [reading research], while others start at eight and go home at five, do what they have always done, and then they retire or go on parental leave. The interest varies a lot. I guess that’s how it is everywhere else too’ (physiotherapist 2, unit 8).

Attitudes and motivation were premised on several factors, including having previous experience with research in one way or another, participation in basic and continuing education and training that involved research issues, as well as being generally curious and keen to learn more to develop as a physiotherapist:

‘I think we are so interested in further training that we do not see it as an obligation. It is more of an opportunity’ (physiotherapist 2, unit 11).

‘We want to treat our patients in the same way. They should get the same tests and treatment regardless of whether they consult with me or anyone else. It [using research] is a kind of quality assurance’ (physiotherapist 3, unit 9).

#### Research-related knowledge and skills

The physiotherapists mentioned that various research-related knowledge and skills were helpful to apply research in clinical practice. One of these competencies was critical or analytical thinking, which they believed facilitated critical appraisal of research studies to determine, for instance, the strength of evidence and whether findings or an approach could be feasible in routine practice:

‘We have made a folder where we have critically appraised all the instruments we use for measuring. Are they really evidence-based? We have also examined if there might be other options, so we are trying to ascertain what is best’ (physiotherapist 1, unit 11).

Several physiotherapists believed qualitative studies are important to obtain a better understanding of many issues, although they regarded physiotherapy research as predominantly quantitative:

‘Qualitative studies may not have the same status, but the ‘soft side’ and other dimensions are starting to be recognized. Also mixed-method studies are emerging. I think this is a positive development’ (physiotherapist 3, unit 6).

#### Workplace level

##### Leadership support

The physiotherapists made it clear that individual managers and leaders played an important role in enabling research use. Many underscored the importance of active encouragement, although the extent to which managers supported research use appeared to vary a great deal:

‘We have had several managers during the years and you notice that they emphasize the importance [of research] differently. Our immediate manager is a paramedic, then there is the manager of the clinic and there are also other people who put pressure on us [to use research results]’ (physiotherapist 1, unit 1).

Some physiotherapists mentioned that their clinics had set goals of improved competence levels for all employees, which contributed to increased research use:

‘It’s not only about the individual; it is about the development of the clinic. If you have been very clinically focused [on patients] for some time, the manager might ask for more efforts that contribute to the development of the whole clinic. It has happened that I have been given the responsibility to investigate or implement new knowledge’ (physiotherapist 1, unit 8).

‘In an ideal world, my ambitions for personal development go hand in hand with the organization’s interest. But it’s the manager’s task to view things in the organization’s best interest, to ensure that there is sufficient breadth of competence in the clinic’ (physiotherapist 2, unit 8).

As well as informal encouragement or support, the physiotherapists also pointed to the importance of formal and explicit management decisions on the desirability of using research in clinical practice. Expectations and strategies concerning research use and a more EBP must be communicated and made clear, according to the physiotherapists:

‘It is absolutely necessary to have the management on your side to get weight behind decisions. Especially since we physiotherapists are often strong-willed individuals, sometimes with disparate ambitions. There has to be a strong leadership behind decisions and the decisions have to be sound’ (physiotherapist 1, unit 8).

#### Organizational culture

The physiotherapists believed that an organizational culture that supports learning and competence in development activities provides favorable circumstances for research use. Although managers’ attitudes and decisions influenced this culture, the physiotherapists also suggested that culture was an independent factor:

‘It’s ingrained, it’s tacit, it’s integrated in the way you work and think in the clinic. It’s about the communication and dialogue, is there space for that kind of discussion and reflection in the clinic?’ (physiotherapist 6, unit 2).

‘It’s important that you work in an environment where you can learn and develop’ (physiotherapist 3, unit 1).

‘We all have different competencies; there is competence breadth. Then there are some who have very narrowly focused competence. But in a positive climate, there are opportunities to learn from those who are very skilled’ (physiotherapist 3, unit 11).

The physiotherapists mentioned that there are higher expectations and demands on them to conduct their own research at a university hospital. They believed this creates a culture in which research is an integral element. Proximity to research competence and participation in ongoing research studies were also mentioned as factors that promote research use for physiotherapists at a university hospital, where research tends to be more integrated in daily practice:

‘I believe it might be different at a university hospital [where] it is more of a tradition and is expected of all professions [to use research]. It’s ingrained in the organization and a way of thinking. You’re closer to ongoing research; patients at a university hospital may be involved in different studies. I believe the use of research is determined by the scope for scientific dialogue’ (physiotherapist 5, unit 2).

#### Research-related resources

The physiotherapists mentioned several types of resources that facilitated research use. An obvious enabler was access to research, including databases that contain research articles. Most physiotherapists had access to such databases. However, they often did not have access to full texts, typically having to rely on abstracts or summaries. This was sometimes solved if a colleague was studying at a university where full-text articles were accessible. Most clinics where the study participants worked subscribed to a physiotherapy journal, usually of a more popular-science nature:

‘*Fysio* [a physiotherapy journal that presents research in a user-friendly format] is not more than 10 pages per issue and it usually includes something about current research, what it [research] says, and everything is summarized, and it’s in Swedish. Then you can go on and dig deeper. It’s very good to have [the journal] here at the clinic’ (physiotherapist 4, unit 3).

Financial and personnel resources also played an important part in giving the physiotherapists opportunities to participate in research-informed courses and conferences and conduct research and development projects:

‘We can apply for funding for [research and development] projects from the County Council. As a first step, you can get funding for two weeks during which you are free to just work with your project idea’ (physiotherapist 4, unit 6).

Financial issues were also deemed important for obtaining any technology that might be required for new research-based treatment approaches:

‘There are costs involved that make it impossible to incorporate new technology that has been found to be effective in research. For example, shockwave is a new thing that has been shown to be effective in research studies, but it’s too expensive so I don’t think we will get it here’ (physiotherapist 3, unit 10).

The physiotherapists also identified time as an important resource that affected their ability to apply research findings in clinical practice. Developing a more EBP approach requires time to identify and appraise research, reflect on its applicability, and apply it in clinical practice:

‘Clinical practice changes take time and energy; it is not possible to just snap your fingers and the change happens. Time and energy and repetition of the change messages are things I believe are necessary to achieve changes in the direction of increased use of research in clinical practice’ (physiotherapist 3, unit 8).

The physiotherapists recognized an obvious conflict between time for production and time for activities that involved learning associated with a more evidence-based physiotherapy practice: ‘In the same breadth that they say that you should take the time to reflect, they mention that you need to see seven patients each day’ (physiotherapist 3, unit 10).

They believed it was necessary to set aside time for individual or group reflection and learning related to EBP: ‘We have regular meetings within the organization, so there are learning opportunities’ (physiotherapist 4, unit 8).

#### Knowledge exchange

The physiotherapists described several forms of knowledge exchange that they believed supported their research use. The discussions that take place with colleagues in the clinic could be both informal, such as everyday conversations about the merits of a specific treatment approach, and more formal with specific meetings devoted to reflection on research studies and new findings and knowledge to facilitate competence development:

‘If someone has participated in a course, they share what they have learned in the course with their colleagues. We allocate one or two hours to that sort of knowledge sharing’ (physiotherapist 4, unit 6).

The physiotherapists reported that their colleagues are the first people they turn to when they need more knowledge or a second opinion about a certain treatment method or to obtain support for testing a new approach. More experienced colleagues are often trusted to have more knowledge about certain patient problems:

‘We cannot continue to treat patients with ineffective methods. If you’re unsure, you ask for a second opinion. We also discuss patients with more complex problems and learn from that. That’s a way to acquire knowledge and implement it’ (physiotherapist 1, unit 11).

The ability to work or collaborate with one or more colleagues with a PhD degree or previous research experience was identified as an important facilitator for research use by some of the physiotherapists. Physiotherapists with a PhD could share information and knowledge about new research and thus provide a resource for colleagues with questions and need for guidance:

‘They [physiotherapists with a PhD degree] are very good to discuss and reflect with. They provide inspiration because they have also been like us, ‘ordinary’ physiotherapists. If you have them [physiotherapists with a PhD degree] in the clinic the ‘distance’ to research doesn’t feel so great’ (physiotherapist 2, unit 6).

Knowledge exchange with clinicians from other professions was also mentioned as an enabling factor for research use. Many of the physiotherapists collaborated with occupational therapists and some worked in multi-professional teams that included nurses and physicians. The physiotherapists believed that this sort of informal inter-professional knowledge exchange contributed to their overall competence development and research use in clinical practice. Mutual trust and respect for one another’s contribution and expertise were critical elements of collaboration that facilitated research use:

‘Implementing something new, as suggested by physiotherapists, depends on how complicated it is to implement it and if they [physicians] believe it’s a good thing. You have to have them ‘on board,’ on your side’ (physiotherapist 2, unit 11).

Knowledge exchange also occurred with patients who might have complex problems or are inquisitive. The physiotherapists noted that today’s patients are generally well-informed about their problems. Many patients have already investigated and done Internet searches so they come prepared for the meeting with the physiotherapist. This was generally found to be motivational and encouraged physiotherapists to keep up with new research findings. Meeting patients with unusual problems also provided a learning opportunity that was supportive of future research use:

‘For the first time, I had a patient suffering from a rare disease. If you know little about what it is you have to do, a lot of reading and learning is necessary to understand the disease and the prognosis. We had a lot of questions and so did he [the patient]. He asked if he was going to get better. To answer that I had to check the statistics of his prognosis. The patient learned a lot from this and I as a physiotherapist did, too’ (physiotherapist 1, unit 7).

#### Extra-organizational level

##### EBP guidelines

The physiotherapists acknowledged that they are expected to adhere to the latest research-based evidence as a basis for best practice. However, they also recognized that the sheer volume of physiotherapy research in the last decade has made it virtually impossible to keep abreast of all new findings. Evidence-based guidelines to assist decisions about appropriate treatment are helpful for research use:

‘National guidelines for stroke and rehabilitation are a way to secure [EBP]. They have done an awful lot of groundwork concerning what is evidence-based and what the recommendations should be. We are encouraged by our manager to form small groups to examine care in terms of stroke and ensure that we work according to the evidence’ (physiotherapist 1, unit 7).

 The physiotherapists made frequent use of the Internet to search for research and check on the guidelines published by the National Board of Health and Welfare (a government department in Sweden under the Ministry of Health and Social Affairs that is responsible for publishing healthcare and social welfare guidelines). They expressed that they wanted to strive towards a more uniform approach to treating their patients although each patient is unique. Guidelines seemed to provide a benchmark from which to start when considering different treatment options:

‘Guidelines make it possible to save time, to go ahead and start treating patients quicker, because it takes time to understand a diagnosis. Guidelines save energy and make work simpler and more effective’ (physiotherapist 6, unit 9).

#### Involvement in external meetings, networks, and conferences

Most of the physiotherapists attended external meetings and/or took part in networks and regional/national conferences at which research is an important topic. They considered this exchange of knowledge and experience with other physiotherapists to be very important for their competence development and commitment to using research in daily clinical practice. Specifically, regularly taking part in conferences was seen as critically important to learn about the latest research developments and findings:

‘Different conferences typically focus on specific topics and have speakers from all over the world talking about the current status concerning that particular research topic; they really explore certain topics at these conferences’ (physiotherapist 4, unit 5).

Some of the physiotherapists had participated in conferences where they presented research and development projects or patient cases.

Informal visits to other clinics were also mentioned as opportunities to learn more and exchange knowledge on research matters with colleagues. Network participation could fulfil similar positive learning and research objectives as conferences:

‘The other week we had a network meeting with physiotherapists from the same region to exchange thoughts. Many things were discussed at these meetings. We feel that we are on the same track’ (physiotherapist 2, unit 9).

These networks could be both formal professional networks and more informal self-established networks comprised of physiotherapists in different clinics from the same region. Some physiotherapists complained that time for engagement in more formal network activities was often limited:

‘Some of the networks are more formal but you often use your personal network if you have are uncertain about how best to treat a patient’ (physiotherapist 2, unit 3).

#### Involvement in academic research and education

Several physiotherapists collaborated with researchers and teachers from nearby universities. Some of the physiotherapists were also engaged in teaching activities and participated in developing curricula for physiotherapy courses. They believed this sort of interaction and involvement contributed positively to their interest in keeping up to date on research and using research as part of their daily practice:

‘Several of us participate in the physiotherapist program, giving lectures and training the students in more hands-on skills. We have also been involved in the examination of students and discussed how the students should be appraised’ (physiotherapist 3, unit 2).

Some of the physiotherapists had participated in research projects led by university researchers and/or taken part in various local research and development projects. Most of the physiotherapists had experience with physiotherapy graduates doing studies and writing their theses at their clinics:

‘I am involved in a research project conducted at the department of physiotherapy at the university, where they are performing an international neck study. I’m working for six weeks with patients who have had neck surgery’ (physiotherapist 3, unit 8).

## Discussion

Nine favorable conditions at three system levels were identified: two conditions at the individual level, four at the workplace level, and three at the extra-organizational level. Conditions at the three levels appear to interact to influence the physiotherapists’ use of research. Hence, physiotherapists are involved in constructing their context, but are in turn influenced by the context, for example, the interpersonal relationships and organizational culture in which they are embedded [43]. Understanding the process of research use in healthcare requires an interdependent, multi-level system perspective, which is echoed in many frameworks and models of implementation, including the Promoting Action on Research Implementation in Health Services (PARIHS) model [32,33,35], the Iowa Model of Evidence-Based Practice [31], the Knowledge-to-Action Framework [34] and the Consolidated Framework for Implementation Research [36].

We found that positive attitudes and motivation to use research, as well as research-related knowledge and skills, provided important individual-level conditions that were perceived as supportive to research use. These factors are likely interdependent, such that research-related knowledge and skills affect attitudes and motivation to use research and vice versa. Attitudes to research have emerged as the single most important factor shaping the use of research among nurses [28]. Findings on determinants for allied health practitioners’ use of research are less consistent; only six studies of relatively weak quality were included in a recent systematic review [44]. Although research has shown that physiotherapists in general are positive to a more EBP, converting these attitudes into changed practice has met with considerable difficulty. The physiotherapists in our study recognized that changing clinical practice is a process that takes time. Several studies have documented that many physiotherapists continue to base practice decisions on knowledge obtained during their initial education and/or personal experience, rather than findings from research [24,45-48]. It has been shown that physiotherapists use treatment techniques with strong or moderate evidence of effectiveness alongside approaches for which evidence is limited or absent [24,45,46,48-50].

The fact that we identified many conditions at the workplace and extra-organizational levels clearly points to the importance of accounting for this influence on the use of research by individual physiotherapists. However, interventions to achieve increased research use in various fields have predominantly targeted individual clinicians [28,29,51]. It is ultimately the individual healthcare professionals who decide whether or not to use research in their practice, which may provide an explanation for the individualized view of research use processes and why many interventions are directed at individuals. However, although research has increasingly recognized the relevance of the workplace or organizational level to research use, Nutley *et al.*[51] believe that knowledge is still lacking on how research might be used at the organizational level and what types of interventions might facilitate increased organizational use of research.

At the workplace level, we identified leadership support, organizational culture, research-related resources, and knowledge exchange as four important conditions that supported the use of research by the physiotherapists, underscoring the significance of achieving an environment that is conducive to the translation of research into practice. Similar to the factors at the individual level, the factors at the organizational level must be considered highly interdependent. For example, a favorable organizational culture is strongly associated with effective leadership in organizations [52-54]. The organizational culture influences how successful leaders are at implementing changes [55,56]. The culture is also related to opportunities for knowledge sharing, learning, reflection, and competence development activities in organizations [57]. Learning, in turn, depends on the availability of some research-related resources, such as time and financial and personnel resources.

The physiotherapists in our study emphasized the importance of formal and informal leadership support for research use. They believed that, to a large extent, research use is a management responsibility, which is consistent with earlier research in various healthcare fields that has shown that healthcare professionals often consider research use to be as much an organizational as an individual responsibility [33,58,59]. Previous physiotherapy research has identified inadequate support from managers as a barrier to research use [19,21,22,60]. Nilsagård and Lohse [22] have proposed that the level of EBP skills (including the ability to find and read research studies, critically appraise evidence, and integrate new findings into their practice) should be considered when recruiting future managers to ensure progression towards more evidence-based physiotherapy. Stevenson *et al.*[19] argue that EBP-skilled opinion leaders, who are not necessarily managers, can be an important influence on other physiotherapists’ commitment to using research. Research in various fields, including healthcare, has shown that opinion leaders—*i.e.*, individuals with specific influence on the attitudes, beliefs, and actions of their colleagues—can indeed be an important strategy to improve the use of research, although opinion leader support alone may not be sufficient to effect practice changes [51].

The physiotherapists believed that an organizational culture that provides opportunities for learning, reflection, and competence development activities facilitated research use. Achieving EBP is reliant on clinicians who acquire EBP skills, that is, the new skills required of today’s physiotherapists (and other healthcare professionals), emphasizing the importance of learning to develop a more EBP. A learning-oriented culture has often been highlighted as a prerequisite for achieving a more EBP in various healthcare fields [61,62]. Similar to our findings, Barnard and Wiles [17] observed that physiotherapists working in university hospitals felt they were part of a research-oriented culture although this was dependent on support from leaders for implementing change and research use. Culture and context are recognized in many of the frameworks and models used in implementation research [63] and in theories concerning concepts such as organizational readiness for change [64] and implementation climate [65]. There is an emerging recognition that findings from organizational and management research can inform implementation research to improve understanding of how the gap between healthcare research and practice can be narrowed [66-68].

Resources such as having access to research studies and sufficient financial and personnel resources and time were identified as important conditions for using research in clinical practice. These factors correspond well with previously identified barriers to physiotherapy research [17-22,26,27]. Lack of sufficient time has almost unanimously been reported as a major hindrance to a more EBP across different healthcare professions. The physiotherapists in our study believed that dedicated time to discuss research was needed. Various solutions have been proposed in the literature, but there appears to be consensus that time must be set aside to provide a formal, scheduled opportunity to meet and discuss relevant research-related matters and that meetings should focus on reflection on research findings and clinical guidelines rather than discussions based on experiential or anecdotal knowledge not linked to research [6,21,69]. However, Heiwe *et al.*[24] have argued that more research is needed into various aspects of the lack of time concept before it is possible to reduce the impact of this factor on implementation of EBP. Limited time is certainly not unique to physiotherapy or healthcare in general, as there is a difficult trade-off between short-term production requirements and longer-term ambitions for learning and development in many work contexts [70].

The physiotherapists in our study stated that knowledge exchange with their physiotherapist peers and colleagues from other healthcare professions supported research use. The importance of peer learning in physiotherapy has been highlighted in previous research on physiotherapists [21] and the lack of peer support and perceived isolation from colleagues have been noted as obstacles to the use of research [21,22,71]. Physiotherapists typically face difficulties when choosing the optimal treatment taking into account the limited evidence base for many of the options, underscoring that peers and colleagues are very important for physiotherapists’ informal learning and their use of research to guide their practice. Knowledge exchange with patients was also found to be conducive to the physiotherapists’ use of research. Patients have been identified in previous research as a key source of knowledge for physiotherapists [26,72]. Physiotherapists listen to the patients’ stories and attempt to understand the context of their life in determining treatment and they collaborate with patients to support regained function and enhance quality of life. Obviously, the holistic nature of much physiotherapy practice does not fit comfortably with the biomedical model of medicine, something that has contributed to considerable debate in the physiotherapy field. Herbert *et al.*[4] succinctly summed up this discussion on physiotherapy when they titled an editorial ‘Evidence-based practice—imperfect but necessary.’

With regard to the extra-organizational level, we identified three conditions that the physiotherapists considered to be supportive of research use: evidence-based guidelines, participation in external meetings, networks, and conferences, as well as involvement in academic research and education. The system level can be seen as an outer context (*i.e.*, factors external to the organization that are related to the wider social, economic, and political context within which organizations reside) that might influence research use via its impact on the workplace and its groups and individuals.

Evidence-based guidelines were seen as supportive to the physiotherapists’ use of research. Clinical practice guidelines are ‘systematically developed statements to assist practitioner and patient decisions about appropriate healthcare for specific clinical circumstances’ [73], a definition adopted by the European Region of the World Confederation for Physical Therapy [74]. By making research findings available to healthcare professionals in a user-friendly format, guidelines are aimed at facilitating EBP. Although physiotherapy has followed the example of other healthcare fields and is producing many guidelines, it lags behind the medical profession in evaluating adherence to and effects of guidelines as well as the effectiveness of various strategies intended to increase their use [75,76].

The physiotherapists believed that various external forums meetings, networks, and conferences were important for research use, which is congruent with research in other fields that has indicated the importance of both formal and informal networks [77]. Recent research has pointed to the critical importance of to healthcare professionals of social networks for the adoption of new practices in healthcare [78,79]. Parchman *et al.*[80] argued that efforts to understand the research–practice gap have been hindered by a lack of recognition of the social networks within which healthcare professionals are embedded. Networks have increasingly emerged as a strategy by governments to facilitate the transfer of more research into clinical practice in healthcare [66].

Involvement in academic research and education was conducive to the physiotherapists’ use of research. Clinical practice and research were interconnected through interaction with colleagues with research experience and with external academic institutions. Our findings lend credence to strategies that have been proposed in various studies, including increased involvement by physiotherapists in research and joint initiatives between academia and healthcare professionals such that students are developing research competence and physiotherapists provide a working laboratory for inquiry [3,69,81,82]. Strategies aimed at strengthening the link between researchers and healthcare professionals as a means to encourage use of research have shown promise in promoting both conceptual and instrumental research use [51]. However, more research is needed to explore how physiotherapists can take part in the research cycle, from planning and conducting studies to the publication, dissemination and implementation of findings.

Several of our results—including the relevance of knowledge exchange with colleagues and patients, interaction with academic institutions, and participation in different external forums—indicate that physiotherapists learn about research through diverse routes. Personal contacts have been found to be an importance source of information about research for professionals in many fields [83,84], and it has been shown that interaction and dialogue can significantly increase the chances that research will be used in various settings [77,85,86].

Our findings suggest that research use in physiotherapy is rarely a simple process of transferring findings from research to practice. It is a complex and dynamic social process that involves a great deal of interaction and knowledge exchange with various people, both internal and external to the workplace. The challenge, according to Greenhalgh *et al.*[87]: [426], is to ‘expose the tensions, map the diversity and communicate the complexity’ to understand the process of using research. The view of research use as an interactive and interpretative social process, rather than as a result of straightforward adoption of research findings, implies that research use is associated with a degree of adaptation of the research itself. This raises the question of whether this process undermines the effectiveness demonstrated by the original research and the extent to which physiotherapy practice can be described as evidence-based. This is an important issue that warrants further investigation.

This study has some shortcomings that must be considered when interpreting the findings. The study was conducted in Sweden and the transferability of the findings beyond the context of the Swedish healthcare system might be limited. Swedish physiotherapists are highly autonomous because they do not depend on referrals from physicians or other healthcare providers, and they can use any physiotherapeutic treatment technique they find suitable. Furthermore, the focus groups may not have been fully representative of all types of physiotherapists in Sweden despite the fact that a heterogeneous purposeful sample was sought.

Research use was not defined by the researcher in the interview situations because the aim was to explore the physiotherapists’ viewpoint of research use. Hence, the physiotherapists had the interpretive prerogative on the meaning of research use because we relied on their subjective interpretation and understanding of research use. They discussed small and large changes due to research, from changes in their understanding and perspectives of issues in physiotherapy to more visible changes in their actual practice, that is, both conceptual and instrumental research use [42].

## Conclusions

We identified nine factors at three interdependent system levels that physiotherapists in Sweden perceived to support their use of research, the individual, workplace, and extra-organizational levels. Research use in physiotherapy appears to be an interactive and interpretative social process that involves considerable interaction with various people, both internal and external to the workplace. The extent to which this process leads to adaptation of the research and affects the effectiveness established in research studies remains unclear.

In terms of clinical implications, this study proposes that interventions to achieve more EBP in physiotherapy through increased use of research in clinical practice must account for a complex interplay between interdependent factors at different system levels. Interventions directed at individual physiotherapists’ skills, knowledge, attitudes, and motivation concerning research use must be considered in a wider context of influences on clinical behaviour. Individually-oriented initiatives for increased research use should be supported by facilitating organizational structures and processes as there is a dynamic interplay between the individual and workplace levels.

## Competing interests

The authors declare that they have no competing interests.

## Authors’ contributions

All authors contributed actively to this paper. PD wrote the first draft, which was discussed with all co-authors, AP and PN. Further drafts were developed in close collaboration among all three authors. All authors approved the final version of the paper.

## Authors’ information

Petra Dannapfel, PhD student, Division of Community Medicine, Department of Medicine and Health, Linköping University, SE-581 83 Linköping, Sweden

Anneli Peolsson, Associate professor, Division of Physiotherapy, Department of Medicine and Health, Linköping University, SE-581 83 Linköping, Sweden

Per Nilsen, Associate professor, Division of Healthcare Analysis, Department of Medicine and Health, Linköping University, SE-581 83 Linköping, Sweden
